# Dominance of toxigenic *Clostridioides difficile* strains and the appearance of the emerging PCR ribotype 955 in hospitals in Silesia, Poland

**DOI:** 10.3389/fmicb.2025.1644051

**Published:** 2025-08-11

**Authors:** Klaudia Szarek, Natalia Frankowska, Monika Kabała, Wiep Klaas Smits, Dorota Wultańska, Piotr Lalowski, Hanna Pituch, Adam Iwanicki, Krzysztof Hinc, Céline Harmanus, Ingrid M. J. G. Sanders, Robert Wojtyczka, Tomasz J. Wąsik

**Affiliations:** ^1^Department of Medical Microbiology, Faculty of Medical Science in Katowice, Medical University of Silesia, Katowice, Poland; ^2^Division of Molecular Bacteriology, Medical University of Gdańsk, Gdańsk, Poland; ^3^Intercollegiate Faculty of Biotechnology, University of Gdańsk, Gdańsk, Poland; ^4^Leiden University Center for Infectious Diseases and Dutch National Expertise Center for Clostridioides difficile, Leiden University Medical Center, Leiden, Netherlands; ^5^Department of Medical Microbiology, Medical University of Warsaw, Warsaw, Poland; ^6^Department of Medical Microbiology, Faculty of Pharmaceutical Sciences in Sosnowiec, Medical University of Silesia, Katowice, Poland

**Keywords:** *Clostridioides difficile*, toxigenic strains, ribotypes, *Clostridioides difficile* infection, hospital-acquired infection, health care-associated CDI

## Abstract

**Introduction:**

Toxigenic strains of *Clostridioides difficile* are the leading cause of healthcare-associated bacterial infections in Poland. In the Silesian Voivodeship, with 4.5 million inhabitants, the incidence of *C. difficile* infection (CDI) in 2023 reached 65.1/100,000, slightly down from 70.0/100,000 in 2022. This study aimed to investigate the occurrence of toxigenic *C. difficile* ribotypes in hospitalized patients in Silesia.

**Materials and methods:**

A total of 130 stool samples from patients with confirmed antibiotic-associated diarrhea were tested. Multiplex PCR detected genes for GDH, toxins A/B, binary toxin CDT, and 16S rDNA. Ribotyping was performed by capillary PCR, and antibiotic susceptibility was tested with ETEST for 10 antibiotics.

**Results:**

Multiplex PCR confirmed the presence of all toxins (A^+^B^+^CDT^+^) in 97 isolates. Six strains (6%) represented ribotype 955 (RT955), first reported in Poland in 2023. RT027 remained dominant (60%, *n* = 78). All isolates were susceptible to vancomycin, while 4% (5/130) showed metronidazole resistance.

**Conclusion:**

RT027 remains prevalent among CDI cases in Silesia. The emergence of RT955, closely related to a UK epidemic strain, suggests a possible shared origin and epidemiological link.

## 1 Introduction

*Clostridioides difficile* is a spore-forming, anaerobic bacterium capable of producing enterotoxin A (*tcdA*), cytotoxin B (*tcdB*), and – in certain strains - an ADP-ribosylotransferase binary toxin (CDT). *C. difficile* infection (CDI) symptoms range from mild antibiotic-associated diarrhea to severe pseudomembranous colitis, toxic megacolon, and sepsis (Hulme, 2024; Alam and Madan, 2024).

The pathogenesis of CDI is associated with the use of broad-spectrum antibiotics (such as fluoroquinolones, clindamycin, third generation cephalosporins, penicillin), age over 65 years and hospitalization (Aptekorz et al., 2017). Spores of *C. difficile* are crucial for the transmission among hospitalized patients (Rawish and Muhammad, 2019; Barbanti and Spigaglia, 2016) remains the main cause of antibiotic-associated diarrhea in hospitalized patients worldwide (National Institute of Public Health, 2025; Mullish and Williams, 2018). *C. difficile* is currently the main factor responsible for outbreaks of bacterial infectious diseases in Polish hospitals (National Institute of Public Health, 2025). According to the reports of the Polish Chief Sanitary Inspectorate in 2023, *C. difficile* was responsible for 362 out of 895 reported outbreaks, while in 2022, *C. difficile* outbreaks accounted for 52% of all registered outbreaks in Poland (2021–53%, 2020–41%, 2019–30%) (National Institute of Public Health, 2025). The incidence of CDI per 100,000 cases in the population from 2021 to 2024 remained relatively constant 2021–55.14; 2022–57.00; 2023–56.06; and for 2024–55.10 (National Institute of Public Health, 2025).

Typing of *C. difficile* isolates by PCR ribotyping, a method based on the detection of heterogeneity in the ribosomal intergenic spacer region (Fawley et al., 2015; Janezic, 2016), indicated the dominance of PCR ribotype (RT) 027 (BI/NAP1/ST1) in Central-Eastern Europe in previous years (Aptekorz et al., 2017; Kuijper et al., 2008). RT027 was first time reported in North America in 2005, and subsequently its spread was observed in Europe and the across the globe (McDonald et al., 2005; Di Bella et al., 2024; Loo et al., 2005). In the years 2008–2010, this type has been detected in 16 countries (Dubberke et al., 2010; National Antibiotic Program, 2025). The RT027 strain was described as capable of producing more toxin A, toxin B, and binary toxin than other strains (Kelly and LaMont, 2008; Curry et al., 2007) is resistant to fluoroquinolones, and is associated with a more severe course of infection (Rawish and Muhammad, 2019; National Antibiotic Program, 2025; He et al., 2013).

Our previous studies from 2017 and 2021 showed that *C. difficile* RT027 dominates in hospitalized patients with CDI in Silesia, Poland, but other ribotypes are also found (Aptekorz et al., 2017; Kabała et al., 2021). Since then, the COVID-19 pandemic has swept the globe, potentially impacting healthcare-associated infections such as *C. difficile* (Rosińska et al., 2024), and several studies have indicated the emergence of new types of *C. difficile* – such as RT176 - in Central Europe (Plankaova et al., 2023; Krutova et al., 2025).

The Silesian Voivodeship in southern Poland, with approximately 4.5 million inhabitants and the highest population density in the country, is a highly urbanized and mobile region bordering the Czech Republic and Slovakia. In 2023, 2,822 cases of CDI were reported in the region, making it one of the most affected areas in Poland (National Institute of Public Health, 2025). Its cross-border movement and high patient flow may contribute to increased pathogen transmission, highlighting the importance of monitoring strain diversity in this setting (Morawiec et al., 2022).

Since data on circulating *C. difficile* ribotypes in Poland after 2021 are lacking, this study addresses an important gap by analyzing the occurrence and characteristics of toxigenic *C. difficile* strains among hospitalized patients in Silesia, a region with a high CDI burden. Understanding the current epidemiology is essential for monitoring the spread of epidemic strains, guiding infection control strategies, and optimizing antimicrobial therapy.

## 2 Materials and methods

One hundred thirty stool samples were collected between March 2023 and March 2024 from hospitalized patients with clinical signs of antibiotic-associated diarrhea. The stool samples were collected in a secondary and a tertiary care hospital localized in Silesia in Southern Poland. One hundred and six samples were obtained from the tertiary care hospital that has 681 beds, 20 wards hospital, and 24 samples were obtained from the secondary care hospital that has 384 beds and 14 wards.

The collected materials were tested for glutamate dehydrogenase (GDH) and *C. difficile* A/B toxins by TechLab C. diff Quick Check Complete (TechLab, Blacksburg, USA), according to a multistep algorithm (National Antibiotic Program, 2025). Samples positive for GDH and toxins were cultured on CDIF-chromID and CLO selective media (bioMérieux, Marcy L’Etoile, France), and the colonies were identified by a VITEK 2 Compact System (bioMérieux, Marcy L’Etoile, France).

Multiplex PCR (mPCR) was performed to detect genes encoding GDH (*gluD*), toxin A (*tcdA*), toxin B (*tcdB*), and bacterial 16S rDNA in *C. difficile* isolates (Persson et al., 2008). Binary toxin (*cdtA*/*cdtB*) genes were detected following the method by Stubbs et al. (2000). The *ermB* gene, conferring MLS_B_ resistance in *C. difficile*, was identified as described earlier (Szarek et al., 2023). PCR products were detected in a 2% agarose gel stained with ethidium bromide (1 mg/L) and analyzed in UV light using the BOX Chemi XR5 (Syngene, UK).

Antibiotic susceptibility of the analyzed isolates was assessed by determining the minimum inhibitory concentration (MIC) of antibiotics using epsilometer tests (ETEST) (bioMérieux, 2025). After 24 h incubation, a few *C. difficile* colonies were suspended in the Schaedler Broth + vit K_3_ to 1 McFarland, then inoculated onto a Brucella Blood Agar plate and ETEST strips were applied (bioMérieux, Marcy L’Etoile, France) for 10 antibiotics: metronidazole - MZ (range 0.016–256 μg/mL), vancomycin – Va (0.016–256 μg/mL), erythromycin – EM (0.016–256 μg/mL), clindamycin – CM (0.016–256 μg/mL), chloramphenicol – CL (0.016–256 μg/mL), moxifloxacin – MX (0.002–32 μg/mL), piperacillin/tazobactam – P/T (0.016–256 μg/mL), benzylpenicillin – PG (0.016–256 μg/mL), imipenem – IP (0.002–32 μg/mL), and rifampicin – RI (0.002–32 μg/mL). The plates were incubated at 37°C for 48 h in an anaerobic chamber (Whitley A35 Workstation, UK). Antibiotic susceptibility results were interpreted according to the EUCAST (European Committee on Antimicrobial Susceptibility Testing) guidelines, version 15.0, effective from January 1, 2025, for Gram-positive anaerobes and *C. difficile* (v.11; 2021) (EUCAST, 2021, 2025). For erythromycin resistance, we assumed a value of ≥ 256 μg/mL.

Ribotyping of *C. difficile* was conducted at Dutch National Expertise Center for *C. difficile* at Leiden University Medical Center in the Netherlands, according to Fawley et al. (2015). Whole genome sequencing of RT955 isolates was performed on an Illumina platform, and analyzed using Seqsphere+ (Ridom) and CSIPhylogeny (Kaas et al., 2014). Whole genome sequence data is available from the sequence read archive via accession PRJNA1291796 with per sample accessions listed in Supplementary Table S1.

## 3 Results

### 3.1 Patient characteristics

In this study, data from 129 patients were analyzed. However, one patient (coded as 20OCH and 24OCH) experienced a recurrence of CDI, confirmed by ribotyping and whole genome sequencing, within a 20-day interval. Consequently, the patient’s readmission was considered a separate hospitalization (*n* = 130). Male patients accounted for 53.8% (*n* = 70). There were no statistical differences in age between male and female patients (*p* > 0.05). The characteristics of the study population are summarized in Table 1.

**Table 1:** Demographic, clinical, epidemiological and analytical data of patients with CDI.

Number of hospitalization	*N* = 130 (*N*=24^a^, *N*=106^b^)
Admission (*N* = 130)	*N*, %
from home	85; 65.4%
another ward or hospital	27; 20.8%
nursing home/senior care facility	10; 7.7%
from a hospice	1; 0.8%
no data	7; 5.4%
Previous hospitalization^c^	111; 85.40%
Previous surgical procedures^c^	57; 43.8%
Length of hospitalization^d^	13 days; [IQR: 8–27]
Time from hospital admission to stool sample collection^d^	4 days [IQR: 1–14]
Comorbidities (*N* = 118*)	*N*; %
hypertension	70; 59.3%
coronary artery disease/ heart failure	63; 53.4%
atherosclerosis	51; 43.2%
diabetes	47; 39.8%
*C. difficile*-associated colitis and enteritis	4; 3.4%
Analytical Data^e^	
creatinine [mg/dL]	1.4 ± 1.17
WBC [×10^9^/L]	14.033 ± 12.231
albumin^f^ [g/L]	24.07 ± 8.67
Previous use of antibiotics	*N*; %
use	92; 70.8%
cephalosporines	38; 41.3%
fluoroquinolones	18; 19.6%
penicillins with beta- lactamase inhibitors	13; 14.1%
no antibiotics	6; 4.6%
no data	32; 24.6%
Antibiotic used for CDI treatment	*N*; %
use	80; 61.5%
vancomycin	55; 68.75%
metronidazole	10; 12.5%
both antibiotics	15; 18.75%
no data	50; 38.5%
Hospital discharge (*N* = 130)	*N*; %
home	94; 72.3%
another ward/ healthcare facility	16; 12.3%
death	18; 13.8%
no data	2; 1.54%

*patients with obtained medical history, re-hospitalized patients were counted only once.

a Secondary care hospital.

b Tertiary care hospital.

c During a 12-week period.

d Median and interquartile range.

e Means ± standard deviation.

f Albumin was measured in only 34 patients.

The hospitals from which the samples were collected differed in both their profiles and the departments where the patients were treated. In 2023, the number of reported CDI cases was 94 in the tertiary care hospital and 76 in the secondary care hospital. The majority of patients at the secondary care hospital were hospitalized in the geriatric ward (8/24), while most patients at the tertiary care hospital were admitted to the Internal and Metabolic Medicine Ward (24/106). Most patients (n = 85; 65.4%) were admitted directly from home, while in 26 patients (30.6%), diarrhea was the primary reason for hospitalization, and stool samples were collected on the day of admission.

Comorbid conditions were present in 91% of patients (118/129), with most patients having more than one comorbidity (*n* = 104; 80.6%). In 11 patients, medical history could not be obtained due to their condition. The patient who was re-hospitalized within 20 days, with no new comorbidities reported, was counted only once. The most frequently reported comorbidities included hypertension (*n* = 70; 59.3%), heart conditions such as coronary artery disease and heart failure (*n* = 63; 53.4%), atherosclerosis (*n* = 51; 43.2%), and diabetes (*n* = 47; 39.8%). *Clostridioides difficile*-associated colitis and enteritis were reported in 4 patients (3.4%). Pseudomembranous colitis was diagnosed in 34 patients (26.2%) during hospitalization, with computed tomography being the primary diagnostic method in most cases (16/34; 47%).

White blood cell (WBC) count and serum creatinine levels were assessed in all patients (*n* = 130). The mean WBC count was 14.033 × 10^9^/L (±12.231), and the mean serum creatinine level was 1.4 mg/dL (±1.17). In contrast, serum albumin concentration was measured in only 34 cases (26.2%), with a mean value of 24.07 g/L (±8.67) in this subgroup.

According to ECDC guidelines, *C. difficile* infections associated with healthcare (HA-CDI) are classified as cases in which symptoms appear more than 48 h after hospital admission or within 12 weeks after discharge from a healthcare facility (ECDC, 2019). Thus, prior hospitalization, surgical procedures, and antibiotic use related to comorbidities are all considered within this 12-week window. Antibiotics were administered to 92/130 patients (70.8%) during hospitalization or the 12 weeks preceding admission, while 6 patients (4.6%) did not receive any antibiotics. The most commonly used antibiotics included cephalosporins (*n* = 38; 41.3%), especially third-generation cephalosporins (*n* = 30; 32.6%), fluoroquinolones (*n* = 18; 19.5%), and penicillins with beta-lactamase inhibitors (*n* = 13; 14.1%). Only two patients were treated with macrolides (*n* = 2; 2.2%), and three patients were treated with lincosamides (*n* = 3; 3.3%).

In the medical history, CDI treatment information was available for only 80 patients. Vancomycin was used in 55/80 (68.75%) cases, while 10/80 (12.5%) received metronidazole, and 15/80 (18.75%) were treated with both vancomycin and metronidazole.

According to the available data, 94 patients (72.3%) were discharged home, while 16 patients (12.3%) required continued care in another ward or healthcare facility. Additionally, 18 patients (13.8%) died, and for 2 patients, no data were available.

### 3.2 Microbiological characterization

All strains (*n* = 130) were susceptible to vancomycin but resistant to moxifloxacin (Table 2); resistance to metronidazole was noted in 5/130 (4%) isolates. Furthermore, 108/130 (83%) *C. difficile* isolates were resistant to benzylpenicillin; almost all strains were resistant to piperacillin-tazobactam (129/130; 99%), imipenem (129/130; 99%), and clindamycin (128/130; 98%). 87/130 of *C. difficile* strains had MIC ≥ 256 for erythromycin. All strains from the secondary hospital with the *ermB* gene were resistant to clindamycin and erythromycin. In the tertiary hospital, MLS_B_ resistance was observed in 41 strains which *ermB* was also detected. 83/130 (64%) isolates were resistant to rifampicin, and 17/130 (13%) to chloramphenicol. The antimicrobial resistance for all tested strains are shown in Supplementary Table S2.

**Table 2:** MIC_50_, MIC_90_ of tested *C. difficile* strains.

Antibiotic	MIC_50_	MIC_90_	Strains resistant		Breakpoints used [mg/L]
			*n* = 130	%	
Metronidazole^a^	1	2	5	4	S ≤ 2, R > 2
Vancomycin^a^	0.38	0.5	0	0	S ≤ 2, R > 2
Erythromycin^b^	256	256	87	67	R > 256
Clindamycin^d^	8	256	128	98	S ≤ 0.25, R > 0.25
Moxifloxacin^c^	32	32	130	100	4
Rifampicin^c^	32	32	83	64	0.004
Piperacillin/Tazobactam^d^	3	6	129	99	S ≤ 0.5, R > 0.5
Imipenem^d^	32	32	129	99	S ≤ 0.5, R > 0.5
Benzylpenicillin^d^	1	2	108	83	S ≤ 0.5, R > 0.5
Chloramphenicol^e^	6	12	17	13	S ≤ 8, R > 8

a Resistance according to EUCAST (v.15 2024).

b Assumed R > 256 mg/L.

c ECOFF for *C. difficile* was used.

d MICs for *C. perfringens* according to EUCAST 2024.

e MICs for Gram-positive anaerobes were used because lack of them according EUCAST (v.11).

**Figure 1:**
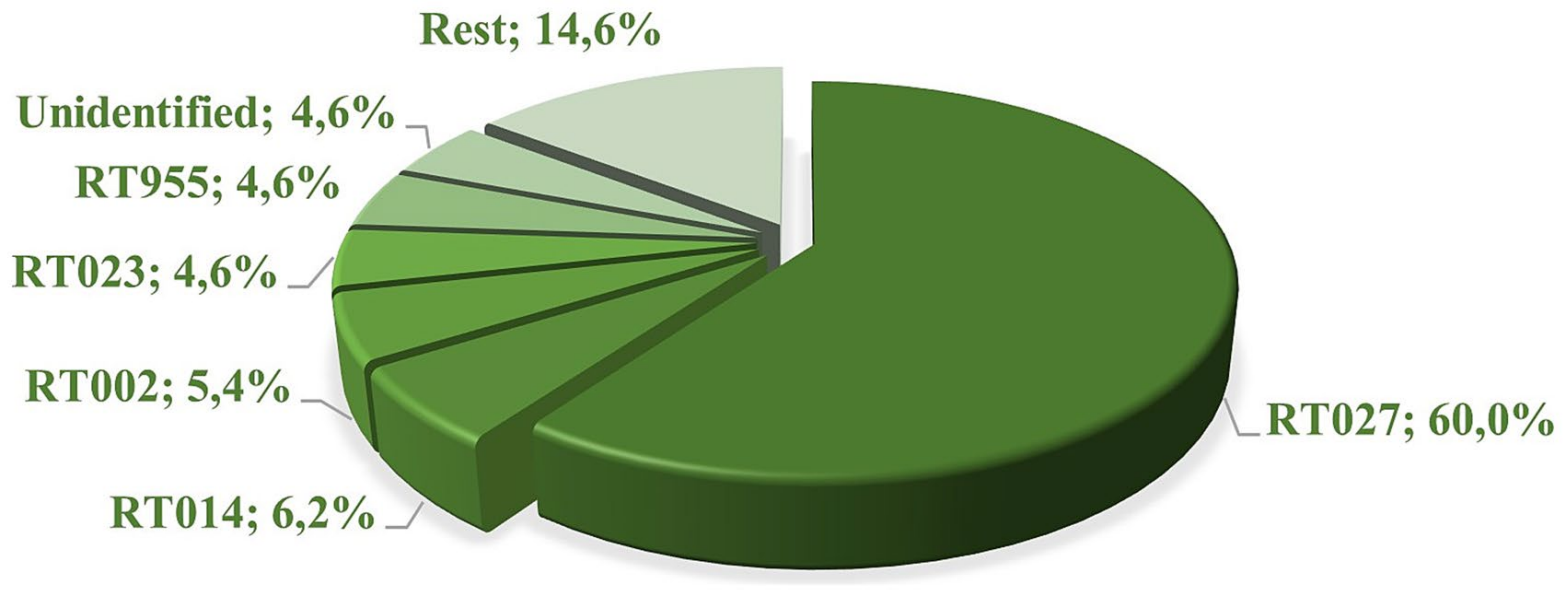
The frequencies [%] of PCR ribotypes in 130 *C. difficile* isolates.

Capillary PCR ribotyping demonstrated a dominance of RT027 (*n* = 78, 60%) (Figure 1), as has also been noted in previous studies in Poland (Aptekorz et al., 2017; He et al., 2013; Persson et al., 2008). Other ribotypes were detected at a 1/10 of this frequency or lower, with RT014 (*n* = 8, 6.2%) RT002 (*n* = 7, 5.4%) and RT023 (*n* = 6, 4.6%). Of note, we also found *n* = 6 isolates belonging to RT955, that has recently been associated with clusters in the United Kingdom (Puleston et al., 2024; South East London, 2024). The remainder of the PCR ribotypes belonged (*n* ≤ 3 for each) to RT018, RT020, RT003, RT005, RT078, RT010, RT011, RT029, RT046, RT070, RT081, and RT430. In six cases, no ribotype could be assigned.

**Figure 2:**
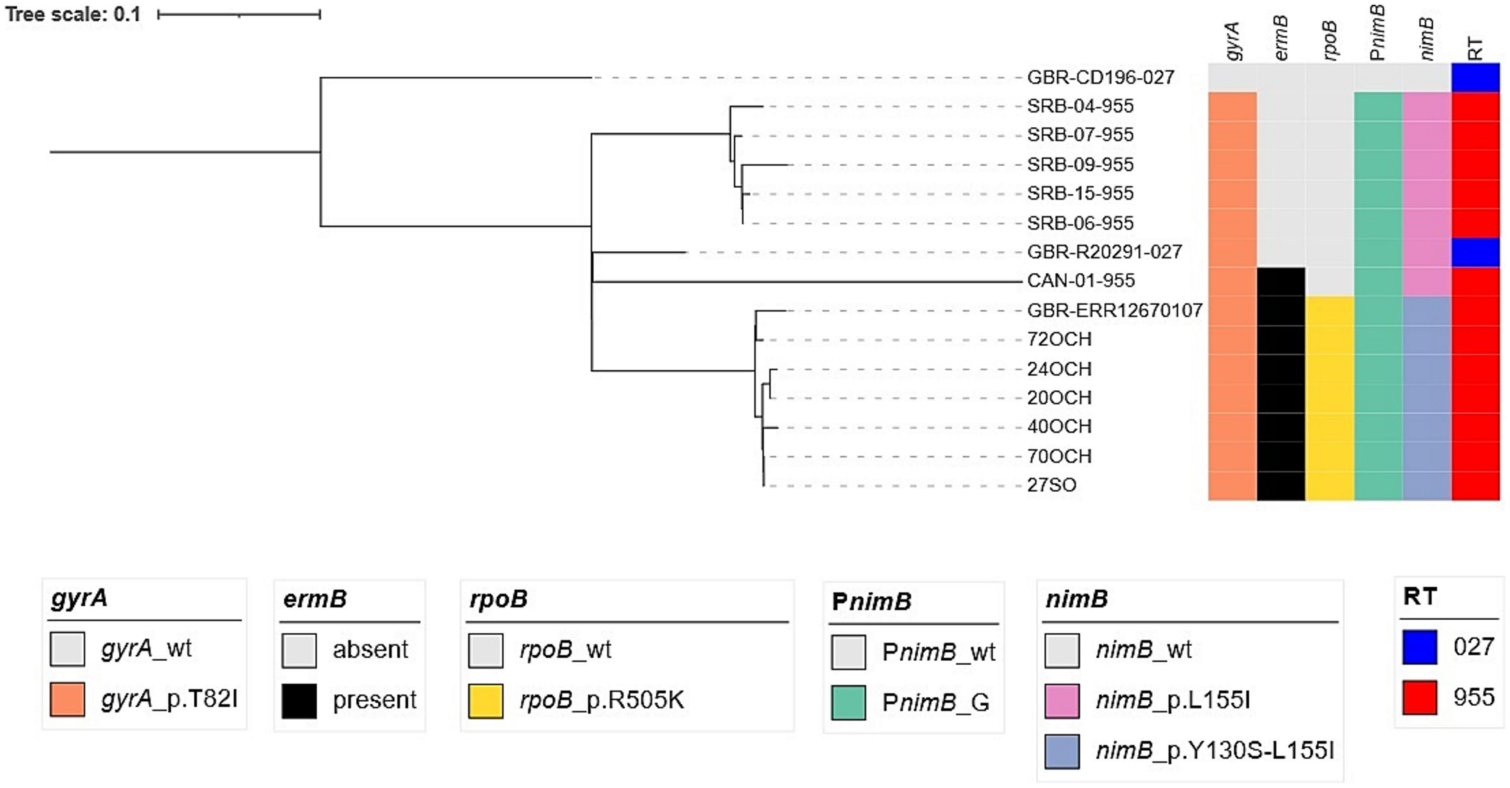
SNP-phylogenetic tree of selected *C. difficile* ST1 isolates (clade 2).

The multiplex PCR reaction demonstrated the A^+^B^+^CDT^+^ genotype in 97 (74.6%) *C. difficile* isolates. Thirty-one strains (23.8%) had the toxigenic profile A^+^B^+^CDT^−^, while the A^−^B^−^CDT^−^ profile was observed in only two strains: one with a ribotype that was not in the database used for identification and the other with ribotype 010. The *ermB* gene, resulting in inducible MLS_B_ type resistance, was detected by PCR in 65 strains (Supplementary Table S1; Figure 2).

Considering the recent interest in this PCR ribotype, the six *C. difficile* isolates identified as RT955 in our study were subjected to whole genome sequencing. They were assigned to multilocus sequence type (ST) 1 and clade 2, a lineage historically associated with the hypervirulent RT027. To evaluate the genetic relatedness to other clade 2 strains belonging to RT955 and RT027, a single nucleotide polymorphism (SNP)-based phylogenetic analysis was performed, using the historical RT027/ST1 reference strain CD196 (GenBank NC_013315) as the root. The resulting phylogenetic tree (Figure 2) includes the following representative isolates for comparison: R20291, the epidemic RT027/ST1 strain associated with the Stoke-Mandeville hospital outbreak in the UK (GenBank NC_013316); a representative of the UK RT955 outbreak strains (GenBank ERR12670107); five unrelated RT955 isolates from Serbia, included from an independent study (Stojanovic et al., 2025); and a Canadian RT955 isolate [genome sequence kindly provided by Tim Du (PHAC/ASPC)]. The SNP analysis clearly demonstrates that the Polish RT955/ST1 isolates form a tight cluster closely related to the UK RT955 outbreak strain ERR12670107, suggesting common ancestry and a possible epidemiological link. Similar to the UK strains, the Polish isolates contain the P*nimB*^G^ mutation, lack the metronidazole-resistance plasmid pCD-METRO, and contain the *gyrA*-p.T82I mutation associated with fluoroquinolone resistance (Plankaova et al., 2023). Moreover, all RT955 contained the *rpoB*-p.R505K mutation associated with rifampin resistance (Supplemental Table S1; Figure 2). In contrast, the Serbian and Canadian RT955 isolates cluster separately, indicating that these strains are genetically distinct from both the UK outbreak and the Polish isolates, despite sharing the RT955 ribotype designation (Figure 2).

## 4 Discussion

Our study showed the dominance of toxigenic strains (97/130; 74.6%) in two hospitals in the Silesian region, many of which belong to ribotypes that have already been displaced in other European countries. This persistent dominance contrasts with a changing trend described in the ECDC report published in May 2024, which analyzed data from 2018 to 2020 across six European countries (Belgium, Estonia, Ireland, the Netherlands, Portugal, and Slovenia) and the UK. According to the report, ribotypes RT014/020, RT002, and RT078 were predominant, while the prevalence of RT027 had significantly declined (ECDC, 2024).

Among the strains analyzed in this study, the RT014 ribotype was recorded in 8/130 (6.2%) strains, while RT002 was present in 7 (5.4%). RT078, which is a ribotype that is also frequently found in in animals and in community-associated cases of CDI in humans, was reported only in two cases. Our data show the dominance of ribotypes capable of producing binary toxin, particularly RT027 (n = 70; 60%), which was in 11th place among the most spread ribotypes in Europe. In our study, no strains closely related to RT027, with the exception of RT955 were reported, consistent with an assessment of the ECDC, that reports that RT027 is mainly found in Poland and neighboring countries (ECDC, 2024).

Interestingly, 6 out of 130 isolates were identified as belonging to the RT955 ribotype, which was detected in Poland for the first time in 2023, in entirely different hospitals across the country (ICDS, 2024; Zikova et al., 2025). One of the RT955 isolates originated from a secondary care hospital, while the remaining five came from a tertiary care hospital — including two isolates from the same patient with a recurrent infection. RT955 came to light in the UK in 2021 as the cause of two large hospital outbreaks (South East London, 2024). In our study, we demonstrated that the Polish RT955 isolates belong to ST1 and are genomically related to the UK strains, but distinct from the Serbian strains (Puleston et al., 2024). RT955 *C. difficile* has been described to be highly transmissible, can lead to severe disease, relapse and shows resistance to treatment with metronidazole commonly used in hospitals (South East London, 2024), but lack of access to clinical and demographic data in this study prevents us from confirming these associations in Poland.

In Poland, fidaxomicin and vancomycin are considered the antibiotics of choice for CDI treatment according to latest recommendation for CDI treatment (National Antibiotic Program, 2025). Our data show that most patients included in our study were treated with vancomycin alone (55 cases) and in 15 cases a combination of vancomycin and metronidazole. However, metronidazole is still commonly used in clinical practice and was used as a single therapy in 10 cases. Fidaxomicin was not available in any of the hospitals included in our study — a limitation that reflects a nationwide issue, not only one specific to the Silesian region. For this reason, we did not assess fidaxomicin susceptibility.

Among the strains we examined, we did not find evidence for resistance to vancomycin, but report 5/130 (4%) strains resistant to metronidazole. Freeman et al. reported that subset of RT955 strains to be metronidazole-resistant when using Fastidious Anaerobe Agar supplemented with horse blood (FAA-HB) in agar dilution experiments (Freeman et al., 2025). In our study, 4/5 metronidazole resistant isolates belonged to RT027 and all RT955 were qualified as susceptible (MIC<4 mg/L). However, 4 out of 6 RT955 strains showed a clearly reduced susceptibility for metronidazole (MIC 2 mg/L). We note that susceptibility testing in our study was performed on BBA medium, which might have led to an underestimation of resistance (Freeman et al., 2025) and on a different media the majority might have been qualified as resistant to metronidazole. On the basis of our whole genome sequence analysis, the reduced susceptibility of the RT955 might be attributable to a previously described mutation in the promoter region of the *nimB* gene (Olaitan et al., 2023). Both RT027 and RT955 represent significant epidemiological threats. In addition to producing all three toxins, they are resistant to fluoroquinolones, which facilitates their spread particularly in hospital settings where these antibiotics are commonly used (Krutova et al., 2014).

Poland is among the countries with the highest antibiotic consumption in Europe, with an average of 23.6 defined daily doses (DDD) per 1,000 inhabitants per day reported in 2022. In contrast, countries like the Netherlands report substantially lower usage, with only 9.1 DDD per 1,000 inhabitants per day (Krotoszyn District Sanitary and Epidemiological Station, 2025). The patients included in our study received broad-spectrum antibiotic therapy due to various comorbidities, as mentioned in the results. The most commonly used group of antibiotics were cephalosporins (38/92; 41.3%), particularly third-generation cephalosporins (30/92; 32.6%). Fluoroquinolones were the second most frequently used group, reported in 18 cases (19.6%). Both of these groups are associated with an increased risk of CDI (Aptekorz et al., 2017).

The Polish law since 2008 regulates, among other things, the functioning of hospital infection control committees. The latest amendment, published on January 1, 2025, introduces requirements such as the implementation of training programs on antibiotic policy in hospital settings. However, our study indicates that despite ongoing efforts, these measures remain insufficient. Although epidemiological surveillance systems are in place, the problem of CDI caused by toxigenic strains appears to be steadily worsening and routine ribotyping and antimicrobial susceptibility testing of *C. difficile* isolates are not yet performed in Poland. As a result, awareness of the actual threat remains low and this may contribute to the emergence of novel PCR ribotypes of concern, such as RT955. Vigilance toward RT027 (in most countries) should be extended to RT955, and in the case of Poland, both should be closely monitored.

## 5 Conclusion

Our study showed the presence of the emerging RT955 ribotype in the Silesia region. The dominance of the toxigenic strains as well as a significant proportion of resistance to metronidazole urgently indicate the necessity to broaden the research on the epidemiology of toxin-producing *C. difficile* strains, with particular focus on the appearance of new ribotypes. The presented data underscore the urgency to include the ribotyping and antimicrobial susceptibility testing of *C. difficile* isolates to the routine diagnostics.

## Data Availability

The datasets presented in this study can be found in online repositories. The names of the repository/repositories and accession number(s) can be found in the article/Supplementary material.
