# Supplementary material for: Dominance of toxigenic Clostridioides difficile strains and the appearance of the emerging PCR ribotype 955 in hospitals in Silesia, Poland
Source: Front Microbiol. 2025 Aug 11;16:1644051. doi: 10.3389/fmicb.2025.1644051 (PMC12376431; doi:10.3389/fmicb.2025.1644051)
Supplement: Supplementary file 1 [file Table_1.DOCX]

Supplementary Material

**Supplementary Table 1S** RT955 *C. difficile* strain GenBank accession numbers

| BioProject | Isolate | Accession | Organism | RT | ST | Country | Isolation date | *gyrA* | *ermB* | *rpoB* | P*nimB* | *nimB* | Reference |
| --- | --- | --- | --- | --- | --- | --- | --- | --- | --- | --- | --- | --- | --- |
| PRJNA1291796 | 20OCH | SAMN49977869 | *C. difficile* | RT955 | ST1 | Poland | March 2023 | T82I | present | R505K | G | Y130S-L155I | This study |
| PRJNA1291796 | 24OCH | SAMN49977870 | *C. difficile* | RT955 | ST1 | Poland | March 2023 | T82I | present | R505K | G | Y130S-L155I | This study |
| PRJNA1291796 | 40OCH | SAMN49977871 | *C. difficile* | RT955 | ST1 | Poland | May 2023 | T82I | present | R505K | G | Y130S-L155I | This study |
| PRJNA1291796 | 70OCH | SAMN49977872 | *C. difficile* | RT955 | ST1 | Poland | November 2023 | T82I | present | R505K | G | Y130S-L155I | This study |
| PRJNA1291796 | 72OCH | SAMN49977873 | *C. difficile* | RT955 | ST1 | Poland | November 2023 | T82I | present | R505K | G | Y130S-L155I | This study |
| PRJNA1291796 | 27SO | SAMN49977874 | *C. difficile* | RT955 | ST1 | Poland | December 2023 | T82I | present | R505K | G | Y130S-L155I | This study |
