# Supplementary material for: Dominance of toxigenic Clostridioides difficile strains and the appearance of the emerging PCR ribotype 955 in hospitals in Silesia, Poland
Source: Front Microbiol. 2025 Aug 11;16:1644051. doi: 10.3389/fmicb.2025.1644051 (PMC12376431; doi:10.3389/fmicb.2025.1644051)
Supplement: Supplementary file 2 [file Table_2.DOCX]

Supplementary Material

**Supplementary Table S2** MIC ranges and PCR ribotypes for tested strains (n=130)

| Strains | Antibiotics | | | | | | | | | | Ribotypes  (RT) |
| --- | --- | --- | --- | --- | --- | --- | --- | --- | --- | --- | --- |
|  | **MZ** | **Va** | **EM** | **CM** | **MX** | **RI** | **P/T** | **IP** | **PG** | **CL** |  |
| 1OCH | <0.16 | 0.38 | 1 | 0.47 | 3 | <0.002 | 2 | >32 | 0.64 | 0.38 | 014 |
| 2OCH | 0.25 | 0.38 | 2 | 6 | 1.5 | 0.004 | 8 | >32 | 1.5 | 6 | 002 |
| 4OCH | 2 | 0.38 | >256 | >256 | >32 | >32 | 8 | >32 | 2 | 12 | 027 |
| 6OCH | 0.125 | 0.38 | 0.75 | 1 | 0.5 | <0.002 | 2 | >32 | 0.094 | 2 | 014 |
| 7OCH | 2 | 0.5 | >256 | 8 | >32 | >32 | 8 | >32 | 2 | 6 | 027 |
| 8OCH | 2 | 0.38 | >256 | 3 | >32 | >32 | 8 | >32 | 1.5 | 4 | 027 |
| 9OCH | 1.5 | 0.38 | >256 | >256 | >32 | >32 | 6 | >32 | 1.5 | 12 | 027 |
| 10OCH | 2 | 0.38 | >256 | >256 | >32 | >32 | 6 | >32 | 1.5 | 8 | 027 |
| 11OCH | 1 | 0.38 | >256 | >256 | >32 | >32 | 4 | >32 | 1.5 | >256 | 027 |
| 12OCH | 1.5 | 0.5 | >256 | 4 | >32 | >32 | 6 | >32 | 1.5 | 4 | 027 |
| 13OCH | 2 | 0.75 | >256 | >256 | >32 | >32 | 6 | >32 | 1.5 | >256 | 027 |
| 14OCH | 1.5 | 0.38 | >256 | 8 | >32 | >32 | 6 | >32 | 1.5 | 4 | 027 |
| 15OCH | 1.5 | 0.5 | >256 | >256 | >32 | >32 | 6 | >32 | 1 | 6 | NR |
| 17OCH | 1 | 0.38 | >256 | 8 | >32 | >32 | 6 | >32 | 1 | 3 | 027 |
| 18OCH | 1.5 | 0.5 | >256 | 6 | >32 | >32 | 12 | >32 | 4 | 4 | 027 |
| 19OCH | 2 | 0.25 | >256 | >256 | >32 | >32 | 8 | >32 | 1.5 | 6 | 027 |
| 20OCH^a^ | 2 | 0.094 | >256 | >256 | >32 | >32 | 4 | >32 | 1.5 | 6 | 955 |
| 21OCH | 1.5 | 0.25 | >256 | >256 | >32 | >32 | 6 | >32 | 2 | 6 | 027 |
| 22OCH | 0.25 | 0.5 | 2 | 2 | 2 | <0.002 | 4 | >32 | 1 | 4 | 014 |
| 23OCH | 1.5 | 0.38 | >256 | >256 | >32 | >32 | 6 | >32 | 1.5 | 6 | 027 |
| 24OCH^a^ | 0.38 | 0.19 | >256 | >256 | >32 | >32 | 3 | >32 | 1.5 | 4 | 955 |
| 25OCH | 1.5 | 0.25 | >256 | >256 | >32 | >32 | 6 | >32 | 1.5 | 4 | 027 |
| 26OCH | 0.25 | 0.38 | 2 | 6 | 4 | <0.002 | 4 | >32 | 1 | 6 | 081 |
| 27OCH | 0.125 | 0.75 | 3 | 8 | 3 | 0.002 | 4 | >32 | 1 | 3 | 003 |
| 28OCH | 1.5 | 1 | >256 | >256 | >32 | >32 | 4 | >32 | 1 | 3 | 027 |
| 29OCH | 1.5 | 0.38 | >256 | >256 | >32 | >32 | 4 | >32 | 1 | 6 | 027 |
| 30OCH | 0.125 | 1 | 2 | 4 | 1 | <0.002 | 6 | >32 | 1.5 | 8 | 023 |
| 31OCH | 1.5 | 0.38 | >256 | >256 | >32 | >32 | 4 | >32 | 1 | 6 | 027 |
| 32OCH | 2 | 0.25 | >256 | 4 | >32 | >32 | 8 | >32 | 4 | 3 | 027 |
| 33OCH | 2 | 0.38 | >256 | 8 | >32 | >32 | 4 | >32 | 1 | 4 | 027 |
| 34OCH | 0.25 | 0.5 | 1.5 | 4 | 2 | <0.002 | 6 | >32 | 1 | 6 | 002 |
| 35OCH | 0.25 | 0.5 | 2 | 4 | 3 | 0.002 | 6 | >32 | 0.5 | 8 | 003 |
| 36OCH | 1.5 | 0.5 | >256 | 8 | >32 | >32 | 12 | >32 | 3 | 6 | 027 |
| 37OCH | 1.5 | 0.5 | >256 | 2 | >32 | >32 | 8 | >32 | 3 | 4 | 027 |
| 38OCH | 1.5 | 0.38 | >256 | 3 | >32 | >32 | 12 | >32 | 3 | 2 | 027 |
| 39OCH | 0.75 | 0.38 | >256 | >256 | >32 | >32 | 4 | >32 | 1 | 4 | 027 |
| 40OCH | 2 | 0.75 | >256 | >256 | >32 | >32 | 3 | >32 | 1 | 8 | 955 |
| 42OCH | 0.5 | 0.75 | 2 | 6 | 1.5 | 0.006 | 4 | 16 | 1 | 8 | 010 |
| 43OCH | 2 | 0.5 | >256 | 4 | >32 | >32 | 6 | >32 | 1.5 | 8 | 027 |
| 44OCH | 2 | 0.38 | >256 | 1.5 | >32 | >32 | 6 | >32 | 1 | 8 | 027 |
| 45OCH | 0.5 | 0.5 | 2 | 3 | 2 | <0.002 | 6 | >32 | 1 | 8 | 020 |
| 46OCH | 2 | 0.25 | >256 | 8 | >32 | >32 | 6 | >32 | 2 | 2 | 027 |
| 47OCH | 2 | 0.38 | >256 | 1.5 | >32 | >32 | 8 | >32 | 2 | 6 | 027 |
| 48OCH | 4 | 0.5 | >256 | 3 | >32 | >32 | 6 | >32 | 1.5 | 6 | N/A |
| 49OCH | 2 | 0.38 | >256 | 2 | >32 | >32 | 8 | >32 | 1.5 | 6 | 027 |
| 50OCH | 0.5 | 0.25 | 0.5 | 1 | >32 | >32 | 4 | >32 | 0.75 | 6 | 027 |
| 51OCH | 0.125 | 0.38 | 0.38 | 1.5 | 0.5 | <0.002 | 3 | >32 | 0.25 | 3 | 005 |
| 52OCH | 0.38 | 1 | 1.5 | 4 | 0.75 | <0.002 | 4 | 4 | 0.39 | 12 | 020 |
| 53OCH | 0.5 | 0.38 | >256 | >256 | >32 | >32 | 4 | >32 | 0.75 | 1 | 027 |
| 54OCH | 0.38 | 0.38 | 0.38 | 0.125 | >32 | >32 | 6 | >32 | 0.75 | 1.5 | 027 |
| 55OCH | 0.064 | 0.5 | 1.5 | 0.047 | 0.5 | <0.002 | 4 | >32 | 0.25 | 2 | 014 |
| 56OCH | 2 | 0.5 | >256 | >256 | >32 | >32 | 4 | >32 | 1 | 4 | 027 |
| 57OCH | 0.75 | 0.5 | >256 | 2 | >32 | >32 | 6 | >32 | 0.75 | 3 | 027 |
| 58OCH | 0.047 | 0.125 | 0.38 | 1.5 | 0.25 | <0.002 | 6 | 4 | 0.75 | 3 | 023 |
| 59OCH | 1 | 0.38 | >256 | 6 | >32 | >32 | 3 | >32 | 0.5 | 4 | 027 |
| 60OCH | 0.25 | 0.38 | 1.5 | 8 | 0.75 | <0.002 | 2 | >32 | 1 | 4 | 430 |
| 61OCH | 1 | 0.19 | >256 | >256 | >32 | >32 | 3 | >32 | 0.75 | 6 | 027 |
| 62OCH | 2 | 0.38 | >256 | 6 | >32 | >32 | 3 | >32 | 1.5 | 4 | 027 |
| 63OCH | 0.75 | 0.38 | >256 | 4 | >32 | >32 | 4 | >32 | 0.75 | 2 | 027 |
| 64OCH | 2 | 0.38 | >256 | >256 | >32 | >32 | 3 | >32 | 1 | 8 | 027 |
| 65OCH | 2 | 0.25 | >256 | 8 | >32 | >32 | 4 | >32 | 1.5 | 8 | 027 |
| 66OCH | 0.75 | 0.5 | 2 | 3 | >32 | <0.002 | 4 | >32 | 1 | 6 | N/A |
| 67OCH | 1.5 | 0.19 | >256 | >256 | >32 | >32 | 3 | >32 | 1 | 8 | 027 |
| 69OCH | 0.19 | 0.25 | 2 | 0.5 | 0.75 | <0.002 | 3 | >32 | 1 | 6 | N/A |
| 70OCH | 2 | 0.25 | >256 | >256 | >32 | >32 | 2 | >32 | 1 | 6 | 955 |
| 71OCH | 1 | 0.25 | >256 | >256 | >32 | >32 | 2 | >32 | 1 | 8 | 027 |
| 72OCH | 1.5 | 0.25 | >256 | >256 | >32 | >32 | 3 | >32 | 1 | 6 | 955 |
| 73OCH | 1.5 | 0.25 | >256 | 3 | >32 | >32 | 3 | >32 | 1 | 6 | 027 |
| 74OCH | 1.5 | 0.19 | >256 | >256 | >32 | >32 | 3 | >32 | 1 | 6 | 027 |
| 75OCH | 0.125 | 0.25 | 1.5 | 12 | >32 | <0.002 | 3 | >32 | 1 | 4 | 078 |
| 76OCH | 0.125 | 0.5 | 1 | 4 | 1 | <0.002 | 4 | >32 | 1 | 4 | 023 |
| 77OCH | 1.5 | 0.25 | >256 | >256 | >32 | >32 | 3 | >32 | 1 | 8 | 027 |
| 78OCH | 0.75 | 0.75 | >256 | >256 | >32 | <0.002 | 3 | >32 | 0.75 | >256 | 027 |
| 79OCH | 3 | 0.19 | >256 | >256 | >32 | >32 | 3 | >32 | 1.5 | 8 | 027 |
| 80OCH | 1.5 | 0.125 | >256 | >256 | >32 | >32 | 3 | >32 | 1 | 3 | 027 |
| 81OCH | 0.75 | 1 | >256 | >256 | >32 | <0.002 | 2 | >32 | 0.75 | >256 | 027 |
| 82OCH | 0.047 | 0.5 | 0.5 | 4 | 0.75 | <0.002 | 4 | >32 | 1 | 6 | 023 |
| 83OCH | 0.5 | 0.5 | 0.75 | 1 | 0.5 | 0.003 | 0.5 | 0.19 | 0.25 | 1.5 | NR |
| 84OCH | 1 | 1.5 | >256 | >256 | >32 | <0.002 | 3 | >32 | 0.75 | >256 | 027 |
| 85OCH | 0.125 | 0.38 | 0.75 | 4 | 1 | <0.002 | 3 | >32 | 0.75 | 4 | 023 |
| 86OCH | 0.125 | 0.19 | 4 | 1 | 0.75 | <0.002 | 2 | >32 | 0.75 | 4 | 014 |
| 87OCH | 1.5 | 0.094 | >256 | >256 | >32 | >32 | 2 | >32 | 1 | 6 | 027 |
| 88OCH | 0.19 | 0.38 | 1 | 8 | 0.75 | <0.002 | 3 | >32 | 0.5 | 4 | 011 |
| 89OCH | 0.125 | 0.25 | >256 | >256 | 1 | <0.002 | 2 | >32 | 1 | >256 | 078 |
| 90OCH | 0.094 | 0.19 | 0.75 | 4 | 1.5 | <0.002 | 2 | >32 | 0.38 | 3 | 018 |
| 91OCH | 0.75 | 0.19 | >256 | 6 | >32 | >32 | 3 | >32 | 0.5 | 2 | 027 |
| 93OCH | 1 | 0.125 | >256 | >256 | >32 | >32 | 2 | >32 | 0.5 | 2 | 027 |
| 94OCH | 0.5 | 0.125 | >256 | >256 | >32 | >32 | 2 | >32 | 0.38 | 6 | 027 |
| 96OCH | 0.75 | 0.094 | >256 | >256 | >32 | >32 | 2 | >32 | 0.38 | 3 | 027 |
| 97OCH | 0.047 | 0.25 | 0.75 | 4 | 2 | <0.002 | 2 | >32 | 0.75 | 3 | 002 |
| 98OCH | 0.094 | 0.25 | 1.5 | 6 | 1.5 | <0.002 | 2 | >32 | 0.5 | 4 | 002 |
| 99OCH | 0.064 | 0.25 | 0.75 | 2 | 1 | <0.002 | 2 | >32 | 0.5 | 3 | 002 |
| 100OCH | 0.094 | 0.25 | >256 | >256 | 0.75 | <0.002 | 1.5 | >32 | 0.38 | 4 | N/A |
| 101OCH | 0.5 | 0.125 | >256 | 3 | >32 | >32 | 2 | >32 | 0.38 | 2 | 027 |
| 102OCH | 2 | 0.19 | >256 | >256 | >32 | >32 | 2 | >32 | 0.38 | 8 | 027 |
| 103OCH | 0.094 | 0.094 | 1 | 1 | 1.5 | <0.002 | 1 | >32 | 0.25 | 3 | 020 |
| 104OCH | 0.064 | 0.125 | 1 | 4 | 0.5 | <0.002 | 2 | >32 | 0.38 | 3 | 014 |
| 105OCH | 0.047 | 0.38 | 0.19 | 1 | 0.75 | <0.002 | 2 | >32 | 0.5 | 2 | 070 |
| 106OCH | 0.064 | 0.064 | >256 | >256 | >32 | >32 | 2 | >32 | 2 | 3 | 027 |
| 107OCH | 2 | 0.19 | >256 | >256 | >32 | >32 | 2 | >32 | 1.5 | 8 | 027 |
| 108OCH | 1 | 0.125 | >256 | >256 | >32 | >32 | 4 | >32 | 2 | 2 | 027 |
| 110OCH | 2 | 0.38 | >256 | >256 | >32 | >32 | 6 | >32 | 3 | 4 | 027 |
| 111OCH | 1.5 | 0.19 | >256 | >256 | >32 | >32 | 3 | >32 | 2 | 8 | 027 |
| 112OCH | 0.5 | 0.5 | >256 | >256 | >32 | >32 | 4 | >32 | 1.5 | >256 | 027 |
| 113OCH | 0.125 | 0.5 | >256 | >256 | >32 | <0.002 | 8 | >32 | 4 | >256 | 046 |
| 114OCH | 0.64 | 0.25 | 1 | 2 | >32 | <0.002 | 1.5 | >32 | 0.38 | 3 | 018 |
| 1SO | 0.125 | 0.25 | 2 | 4 | >32 | <0.02 | 3 | >32 | 1.5 | 3 | 002 |
| 2SO | 0.125 | 0.25 | 1.5 | 4 | >32 | <0.02 | 2 | >32 | 1.5 | 3 | 014 |
| 3SO | 1.5 | 0.125 | >256 | >256 | >32 | >32 | 2 | >32 | 0.75 | >256 | 027 |
| 4SO | 0.19 | 0.25 | 2 | 4 | >32 | <0.02 | 3 | >32 | 1 | 4 | 002 |
| 6SO | 0.19 | 1 | >256 | >256 | >32 | >32 | 6 | >32 | 1.5 | 3 | 027 |
| 7SO | 3 | 0.25 | >256 | >256 | >32 | >32 | 3 | >32 | 1.5 | 6 | 027 |
| 8SO | 2 | 0.19 | >256 | >256 | >32 | >32 | 3 | >32 | 1.5 | >256 | 027 |
| 9SO | 0.125 | 1 | 2 | 2 | 1 | 0.12 | 4 | >32 | 1 | 3 | 005 |
| 10SO | 1 | 0.19 | >256 | >256 | >32 | >32 | 3 | >32 | 0.75 | >256 | 027 |
| 11SO | 4 | 0.19 | >256 | >256 | >32 | >32 | 2 | >32 | 1.5 | 6 | 027 |
| 12SO | 2 | 0.25 | >256 | >256 | >32 | >32 | 3 | >32 | 1 | 6 | 027 |
| 14SO | 2 | 0.19 | >256 | >256 | >32 | >32 | 3 | >32 | 1 | >256 | 027 |
| 15SO | 2 | 0.19 | >256 | >256 | >32 | >32 | 2 | >32 | 1.5 | 4 | 027 |
| 16SO | 2 | 0.25 | >256 | >256 | >32 | >32 | 3 | >32 | 1.5 | 8 | 027 |
| 17SO | 0.125 | 0.25 | 0.75 | 3 | 1.5 | <0.02 | 3 | >32 | 0.75 | 3 | 018 |
| 21SO | 0.38 | 0.5 | 1.5 | 6 | 1.5 | <0.02 | 4 | >32 | >32 | 16 | 029 |
| 22SO | 4 | 0.25 | >256 | >256 | >32 | >32 | 3 | >32 | 1.5 | 6 | 027 |
| 23SO | 2 | 0.25 | >256 | >256 | >32 | >32 | 4 | >32 | 0.75 | 3 | 027 |
| 25SO | 2 | 0.25 | >256 | >256 | >32 | >32 | 2 | >32 | 1.5 | 4 | 027 |
| 26SO | 1.5 | 0.38 | >256 | >256 | >32 | >32 | 4 | >32 | 1 | 3 | 027 |
| 27SO | 2 | 0.38 | >256 | >256 | >32 | >32 | 3 | >32 | 1 | 4 | 955 |
| 28SO | 0.125 | 0.5 | 1.5 | 12 | 1 | <0.02 | 4 | >32 | 1.5 | 12 | 023 |
| 30SO | 0.19 | 0.38 | 1.5 | 4 | 2 | <0.02 | 2 | >32 | 1 | 6 | 014 |
| 31SO | 1 | 0.125 | >256 | >256 | >32 | >32 | 2 | >32 | 1 | 4 | 027 |

^a^ Strains isolated from the same patient

N/A - not in the database

NR - not recovered for PCR ribotyping
